# Memory performance in the preclinical stage of ADAD: The influence of APOE ε4 on *PSEN1_A431E_* and *APP_V717I_* Carriers

**DOI:** 10.1002/alz70855_102487

**Published:** 2025-12-23

**Authors:** Frida Rosales‐Leycegui, Angélica Zuno‐Reyes, César A Valdez‐Gaxiola, Ana Karen Preciado‐Baron, Isaac Enrique Berumen‐Ocegueda, Karina Pérez‐Rubio, Sofia Dumois‐Petersen, Ricardo Jáuregui Torres, Luis E Figuera, John M Ringman, Esmeralda Matute

**Affiliations:** ^1^ Instituto de Neurociencias, CUCBA, Universidad de Guadalajara, Guadalajara, JA, Mexico; ^2^ Universidad de Guadalajara, Guadalajara, JA, Mexico; ^3^ Instituto de Neurociencias, Universidad de Guadalajara, Guadalajara, JA, Mexico; ^4^ Instituto Mexicano del Seguro Social, Guadalajara, JA, Mexico; ^5^ Alzheimer's Disease Research Center, Keck School of Medicine, University of Southern California, Los Angeles, CA, USA

## Abstract

**Background:**

Autosomal dominant Alzheimer's disease (ADAD) provides a unique model to study early cognitive changes associated with Alzheimer's disease (AD) pathological hallmarks. Although *APOE ε4* is the main genetic risk factor for sporadic AD, related to the apparition of amnestic phenotypes, its relationship with cognitive features in ADAD remains unclear. This study investigates the influence of the co‐occurrence of APOE ε4 with *APP_V717I_* or *PSEN1_A431E_* mutations on cognitive performance in the preclinical phase of ADAD.

**Method:**

A total of 81 Mexican‐mestizo individuals at risk of inheriting one of these mutations, underwent genetic analysis, including *PSEN1_A431E_/APP_V717I_* mutation screening and *APOE* genotyping, as well as neuropsychological evaluation using the CERAD‐MX battery. Based on genetic results, participants were divided into carriers (C; *n* = 39 [14 *APP_V717I_* and 25 *PSEN1_A431E_*]) and non‐carriers (NC; *n* = 42). Further stratification by *APOE* genotype (ε4+ or ε4‐) resulted in four subgroups: ε4+/ε4‐ carriers (Cε4+ *n* = 6 /Cε4‐ *n* = 33), and ε4+/ε4‐ non‐carriers (NCε4+ *n* = 5 /NCε4‐ *n* = 37). Normalized z‐scores from 12 tasks of the CERAD‐MX were taken as measures of global cognition, memory, language, attention, visuospatial skills, and executive functions. The Clinical Dementia Rating (CDR) was used to determine preclinical staging. Group differences were analyzed using Mann‐Whitney U, Kruskal‐Wallis, and Dunn's post hoc tests.

**Result:**

Comparisons revealed that mutation carriers exhibited lower performance in Constructional Praxis‐Recall (NC>C, *U* = 582, *p* = .02) and Rey‐Osterrieth Complex Figure‐Recall (RCF; NC>C *U* = 503, *p* = .002) visual memory tasks. When stratified by *APOE*, Cε4‐ participants showed a significant difference in RCF‐recall compared to NCε4‐ (*k* = 10.32, *p* = .009).

**Conclusion:**

Our findings indicate early visual memory impairment preceding the ADAD clinical stage in this carrier population. However, no evidence was found to indicate a negative impact of *APOE ε4* on memory performance. Future efforts to expand the sample size will be essential to confirm whether the observed pattern persists and to uncover potential effects on other cognitive domains. Additionally, factors such as proximity to symptom onset and *APOE* ancestry may also influence cognitive performance. This study contributes to understanding early cognitive changes in ADAD and suggests that factors modifying the clinical phenotype may also play a role in shaping preclinical cognitive variations.

